# Two novel alleles of the MYB transcription factor *BjA06.GL1* and *BjB02.GL1* control leaf trichomes and enhance resistance to aphids in *Brassica juncea*

**DOI:** 10.1093/hr/uhae314

**Published:** 2024-11-12

**Authors:** Shuangping Heng, Xiaolin Li, Man Li, Lulu Jiang, Meng Li, Wei Zeng, Guangzhi Mao, Feng Xing, Zhengjie Wan, Jing Wen, Jinxiong Shen, Tingdong Fu

**Affiliations:** College of Life Science, Xinyang Normal University, No. 237 Nauhu Road, Changan District, Xinyang 464000, China; College of Life Science, Xinyang Normal University, No. 237 Nauhu Road, Changan District, Xinyang 464000, China; College of Life Science, Xinyang Normal University, No. 237 Nauhu Road, Changan District, Xinyang 464000, China; College of Life Science, Xinyang Normal University, No. 237 Nauhu Road, Changan District, Xinyang 464000, China; College of Life Science, Xinyang Normal University, No. 237 Nauhu Road, Changan District, Xinyang 464000, China; College of Life Science, Xinyang Normal University, No. 237 Nauhu Road, Changan District, Xinyang 464000, China; College of Life Science, Xinyang Normal University, No. 237 Nauhu Road, Changan District, Xinyang 464000, China; College of Life Science, Xinyang Normal University, No. 237 Nauhu Road, Changan District, Xinyang 464000, China; National Key Laboratory of Germplasm Innovation & Utilization of Horticultural Crops, College of Horticulture and Forestry, Huazhong Agricultural University, No.1 Shizishan Street, Hongshan District, Wuhan 430070, China; National Key Laboratory of Crop Genetic Improvement, College of Plant Science and Technology, Huazhong Agricultural University, No.1 Shizishan Street, Hongshan District, Wuhan 430070, China; National Key Laboratory of Crop Genetic Improvement, College of Plant Science and Technology, Huazhong Agricultural University, No.1 Shizishan Street, Hongshan District, Wuhan 430070, China; National Key Laboratory of Crop Genetic Improvement, College of Plant Science and Technology, Huazhong Agricultural University, No.1 Shizishan Street, Hongshan District, Wuhan 430070, China

## Abstract

Leaf trichome formation is a very important agronomic trait as it confers resistance to biotic and abiotic stresses, but the causal genes involved in this process in *Brassica juncea* remain largely unexplored. In this study, we first characterized the haplotypes of *BjB02.GL1* among different inbred lines with leaf trichomes or glabrous leaves. A comparative analysis of the number and density of leaf trichomes between the two mustard inbred lines was then performed*.* BSA analysis of leaves with trichomes and glabrous pools from the F2 segregating population mapped the candidate genes on Chr.A06 and Chr.B02. Two candidate genes, *BjA06.GL1* and *BjB02.GL1*, were subsequently cloned. After sequence alignment of the *BjGL1* genes, both single-nucleotide polymorphisms (SNPs) and indel were identified in the *BjA06.GL1* and *BjB02.GL1* genes. And quantitative real-time polymerase chain reaction (qRT-PCR) analysis further confirmed that both the *BjA06.GL1* and *BjB02.GL1* genes were more highly expressed in leaves with trichomes than in glabrous leaves. As the leaf size increased, the leaf trichome density decreased. Gene editing of both *BjA06.GL1* and *BjB02.GL1* changed the leaf trichome to a glabrous leaf phenotype in mustard. In addition, plants with leaf trichomes presented greater resistance to aphids. Taken together, our results revealed that both *BjA06.GL1* and *BjB02.GL1* positively regulate leaf trichome formation and help increase aphid resistance in mustard. This study provides valuable resources and helps to elucidate the molecular mechanism of leaf trichome formation in *B. juncea*.

## Introduction

Mustard (*Brassica juncea*) is widely cultivated as an important oil crop and is also consumed as a fresh or pickled vegetable [1]. Allotetraploid mustard is formed by natural hybridization of *Brassica rapa* and *Brassica nigra* followed by natural doubling [2]. In mustard, trichomes are single-celled, nonbranched, and nonglandular. The presence of leaf trichomes affects the response to its biotic and abiotic stresses. Both leaf trichomes and glucosinolates increase quickly after herbivory-related damage [3]. Leaf trichome density increases on both adaxial and abaxial leaf surfaces in response to herbivory [4]. The nonbranched leaf trichomes in *B. juncea* appear only in the vegetative stage and disappear in the reproductive stage [5]. This is apparently different from the observations for the model plant *Arabidopsis*. Nevertheless, leaf trichome formation in *B. juncea* is complex and involves orchestrated regulatory processes.

Plant trichomes are cellular structures that typically appear as elongated protrusions and can be found on different plant organs, such as roots, stems, leaves, and flowers [6]. On the basis of their diverse morphologies and structures, trichomes can be further categorized as unicellular or multicellular, branched or nonbranched, glandular or nonglandular among different plant species [7–9]. Trichomes perform multiple functions in plants, playing important roles in biotic and abiotic stress resistance [10]. Trichomes can absorb and excrete substances, assisting plants in regulating water and nutrient balance [11]. Trichomes can also synthesize and store specialized metabolites, such as volatile organic compounds and secondary metabolites, which play important roles in plant physiology and ecological adaptation [12]. The molecular mechanisms underlying the functions of trichomes involve the synthesis and transport of specialized metabolites, as well as the regulation of signaling pathways. The regulation of signaling pathways involves the participation of hormones, transcription factors, and signaling molecules, which regulate the process of trichome formation [12, 13]. *Arabidopsis* has been widely used as a well-studied model for elucidating leaf trichome development and differentiation. A trimeric complex, MYB-bHLH-TTG, forms a core gene regulatory network to activate leaf trichomes [14, 15]. Some negative regulators, including AtCPC, AtTRY, AtETC1, and AtETC2, can interact with AtGL3, AtEGL3, and AtTTG1 to negatively regulate leaf trichome development [16].

The functional study of trichomes in plants has provided valuable insights into the molecular mechanisms of trichome development and their roles in plant physiology and defense. In recent years, an increasing number of studies have focused on the molecular mechanisms underlying trichome function. GL1 encodes an MYB transcriptional regulator, and *GL1* is among the central hub genes involved in the production of trichomes [5, 17]. In *Arabidopsis*, recessive mutation of *AtGL1* prevents the initiation of leaf trichomes, and overexpression of AtGL1 inhibits trichome development [18]. AtTOE1, which encodes an AP2-like transcription factor, can bind to the 3′ noncoding region of *AtGL1* to regulate abaxial trichome initiation [19]. The functional *AtGL1* gene significantly increased *Arabidopsis* resistance to flea beetles and turnip sawflies under field growth conditions [20]. Both RrGL1 from *Rosa roxburghii* and BnaA.GL1.a from *Brassica napus* could functionally complement the *Arabidopsis* gl1 recessive mutation to regulate trichome initiation [21, 22]. A unique allele of the Chinese cabbage gene *BrTRI1*, which is homologous to *AtGL1*, regulates the variation in trichome formation [23]. In radish, either or both functional genes *RsGL1a* and *RsGL1b* contribute to the leaf trichome formation [24]. A previous study revealed that a 3-kb structural variation located on *GL1* (BjuVB02G54610) is strongly associated with leaf trichomes in *B. juncea* [5]. In the *Brassicaceae* family, the *GL1* homologous genes exhibit differential effects on trichome patterning. Further exploration of key genes involved in leaf trichome formation in mustard is needed to elucidate the genetic variation in leaf trichomes.

In this study, the genetic basis of leaf trichome formation in *B. juncea* was further dissected. In addition to the *BjB02.GL1* gene located on Chr.B02, the *BjA06.GL1* gene located on Chr.A06, which encodes the R2R3 MYB transcription factor, was also identified. After a comparative analysis of the *BjA06.GL1* and *BjB02.GL1* genes, Indel and single-nucleotide polymorphisms (SNPs) were found to exist in the *BjA06.GL1* and *BjB02.GL1* genes of H2 and H177*.* The *BjA06.GL1* and *BjB02.GL1* genes were highly homologous, and the similarity of their putative amino acid sequences was 90.52%. Transcript expression levels of the *BjA06.GL1* and *BjB02.GL1* genes in leaves with trichomes was significantly greater than those in glabrous leaves. CRISPR/Cas9-mediated editing of the *BjA06.GL1* and *BjB02.GL1* genes led to the formation of glabrous leaves. The leaf trichomes of mustard showed strong resistance to aphids. This study not only provides new insight into leaf trichome formation, but also provides valuable resources for breeding vegetable mustards.

## Results

### LTR insertion in *BjB02.GL1* gene was not fully associated with leaf trichomes

**Figure 1:**
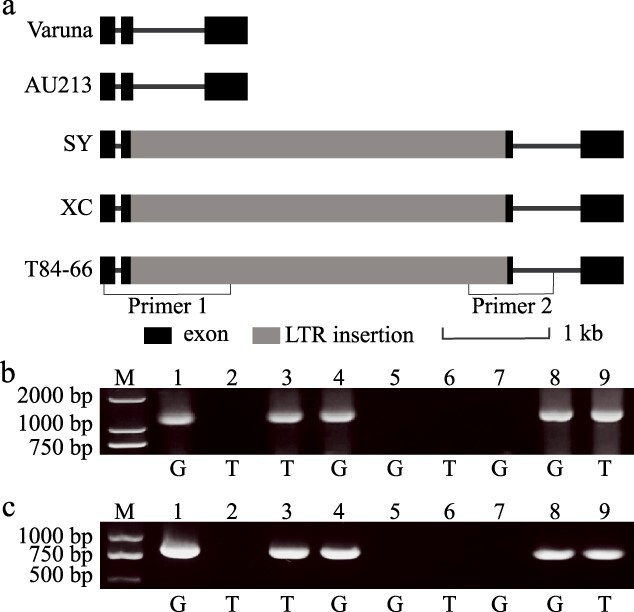
Haplotype of the *BjB02.GL1* gene among different mustard lines with leaf trichomes or glabrous leaves. a. Gene structural variation of the *BjB02.GL1* gene among five sequenced mustard genomes. b. Haplotype of the *BjB02.GL1* detected with primer 1 showed that the LTR insertion was not related to the presence or absence of leaf trichomes. c. Haplotype of the *BjB02.GL1* detected with primer 2 showed that the LTR insertion was not related to the presence or absence of leaf trichomes. Lanes 1–9 represent different mustard inbred lines (XC, 102B, H58, H10, H177, H2, Varuna, SY, and T84–66) with glabrous leaves or leaf trichomes. G: mustard inbred line with glabrous leaves, T: mustard inbred line with leaf trichomes. The label ‘M’ represents the DNA molecular marker, and the numbers represent the DNA molecular weights.

A previous study suggested that the 3-kb structural variation (SV) located on BjuVB02G54610 was strongly associated with leaf trichome formation [5]. Here, we first compared the *BjB02.GL1* gene from the five sequenced *B. juncea* strains, including three oil types and two vegetable-used mustard lines (Fig. 1a). A 3791-bp insertion was found in the second exon of *BjB02.GL1* gene from one oil-type mustard (BjuB02g57140s, SY) and two vegetable-type mustards (BjuVB02G54610, T84–66 and BjuLB02G52550, XC). No insertions were found in the *BjB02.GL1* gene from the oil-type mustard (BjuOB02G58240, AU213, and BjuB08_VARUNA_g2160, Varuna). Blast analysis indicated that the insertion was a copia LTR retrotransposon. Two molecular markers related to the LTR insertion were subsequently developed to identify the relationships between the SV and leaf trichomes in *B. juncea*. Interestingly, inbred lines (H177 and Varuna) with glabrous leaves did not exhibit the LTR insertion in their *BjB02.GL1* genes. However, the inbred lines (H58 and T84–66) with leaf trichomes exhibited the LTR insertion in their *BjB02.GL1* genes (Fig. 1b, c). Obviously, the LTR insertion in the *BjB02.GL1* gene was not fully associated with leaf trichomes. Therefore, other genes may be involved in regulating leaf trichome formation in *B. juncea*.

### Phenotypic and genetic analysis of leaf trichomes of H2 and H177 in *B. juncea*

**Figure 2:**
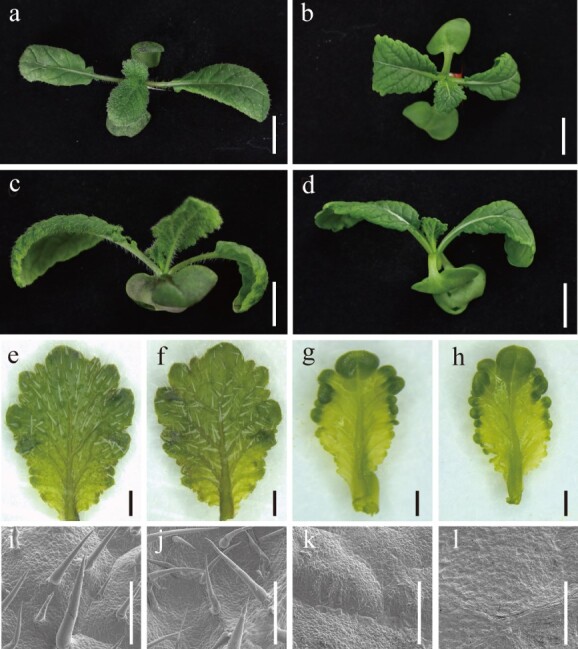
Phenotypes of mustard leaves from the two parent lines with leaf trichomes or glabrous leaves. a. Top view of leaf trichomes of the parent line H2. b. Top view of glabrous leaves of the parent line H177. c. Elevation view of the leaf trichomes of the parent line H2. d. Elevation view of glabrous leaves of the parent line H177. e. Adaxial epidermal leaf from H2. f. Abaxial epidermal leaf from H2. g. Adaxial epidermal leaf from H177. h. Abaxial epidermal leaf from H177. i. SEM analysis of the adaxial epidermal leaf from H2. j. SEM analysis of the abaxial epidermal leaf from H2. k. SEM analysis of the adaxial epidermal leaf from H177. l. SEM analysis of the abaxial epidermal leaf from H177. a–d Scale bars = 1 cm; e–h Scale bars =1 mm; i–l Scale bars = 500 μm.

To further understand the genetics of the leaf trichomes, H2, with leaf trichomes, and H177, with glabrous leaves, were selected. Trichomes appeared on both the adaxial epidermal leaves (Fig. 2a, e) and abaxial epidermal leaves (Fig. 2c, f) of H2. However, the adaxial (Fig. 2b, g) and abaxial (Fig. 2d, h) epidermal leaves on H177 were glabrous. Scanning electron microscopy (SEM) analysis revealed that the leaf trichomes from H2 were single cell and nonbranched (Fig. 2i, j). Leaf trichomes were rarely observed in H177 (Fig. 2k, l). As the number of leaves increased, the number of trichomes on the H2 leaves first increased, but then decreased (Fig. S1a). However, the density of trichomes on the H2 leaves decreased (Fig. S1b). The *B. juncea* inbred lines H2 and H177 were then used as the parent lines to further construct the segregated population. Leaf trichomes were present in the F1 mustard leaves, which indicates that the leaf trichome phenotype was the dominant trait over the glabrous phenotype in *B. juncea*. In the F2 segregation population with 1585 plants, the segregation ratio between leaf trichomes and glabrous phenotypes was 10:1 (1444:141). These results revealed that the leaf trichome phenotype in mustard is a complex quantitative trait.

### Rapid identification of candidate genes for leaf trichomes in *B. juncea*

**Figure 3:**
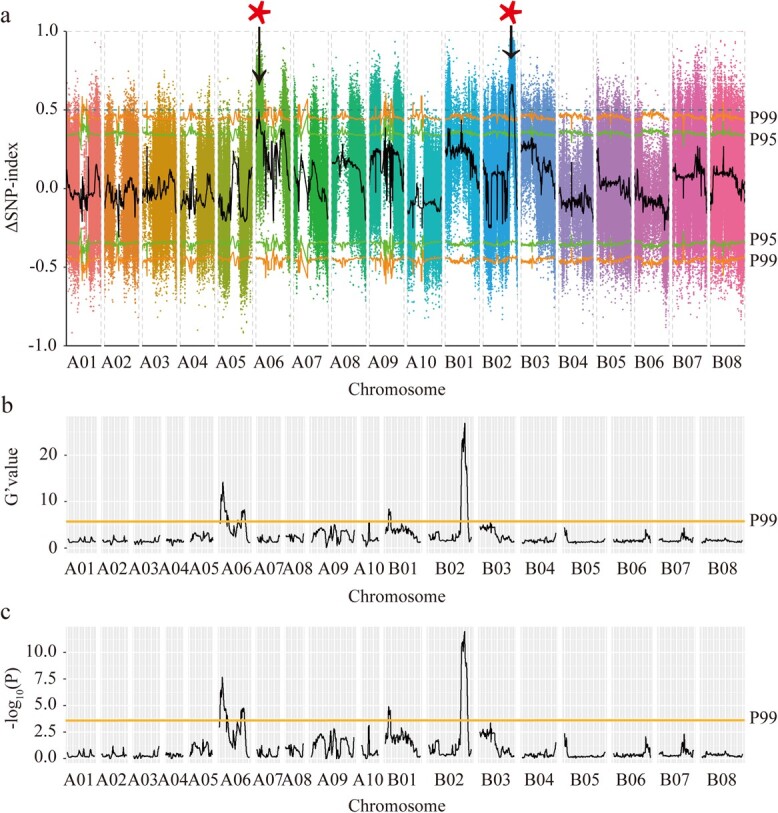
QTL-seq analysis of quantitative trait loci related to leaf trichome formation. a. Manhattan plot showing the distribution of the ΔSNP index across chromosomes. b. Manhattan plot showing the distribution of the G’ values across chromosomes. c. Manhattan plot showing the distribution of the -log_10_ (*P-*value) value on the chromosomes based on Fisher’s exact test. The P95 and P99 represent the 95% and 99% confidence levels, respectively.

Quantitative trait loci (QTL)-seq analysis was used for preliminary mapping of the genetic region related to leaf trichomes. First, 30 plants with leaf trichomes were selected for the trichome pool, and 30 plants with glabrous leaves were selected for the glabrous pool from the F2 segregation population. A total of 117.5 Gb of clean resequencing data were generated from the two parent lines, H2 and H177, the trichome pool and the glabrous pool, respectively (Table S1). More than 98.9% of the clean reads were mapped to the leaf mustard XC genome. A total of 2 174 067 SNPs were identified between the trichome pool and the glabrous pool. The locations of the candidate genes for leaf trichomes were preliminarily mapped to 3.9–6 Mb on Chr.A06 and 55.2–66.9 Mb on Chr.B02 via the Δ(SNP index) algorithm at the 99% significance level (Fig. 3a). The locations of the candidate genes were further mapped to 4.6–14.1 Mb, 30.4 Mb, and 36.6–43.4 Mb on Chr.A06; 4.6–8.7 Mb on Chr.B01; 55.2–66.9 Mb on Chr.B02; and 18.1–18.2 Mb on Chr.B03 on the basis of the G’ value and Fisher’s exact test (Fig. 3b and c). According to the overlap interval of the three algorithm models, the candidate genes were narrowed to 4.6–6 Mb on Chr.A06 and 55.2–66.9 Mb on Chr.B02. On the basis of the highly credible SNPs in the genes, 239 and 1207 genes (Table S2, Table S3) were identified in the two candidate chromosome regions. Gene function analysis revealed that only the *BjA06.GL1* (BjuLA06G007240) and *BjB02.GL1* (BjuLB02G052550) genes, which are homologous to *AtGL1*, may be responsible for leaf trichome formation. Molecular markers were subsequently developed to identify the *BjA06.GL1* and *BjB02.GL1* genes between the two parental lines with and without trichomes. The difference in the *BjA06.GL1* and *BjB02.GL1* genes between the two parent lines explained 78.87% and 87.32% of the leaf trichome variation in the F2 segregating population (Fig. S2). However, when combined with *BjA06.GL1* and *BjB02.GL1*, >97.18% of the variation in the F2 segregating population could be explained. The two molecular markers could explain leaf trichome variation in 4 other inbred lines (H177, Varuna, H58, and T84–66), which could not be explained by the LTR insertion in the *BjB02.GL1* gene (Fig. S3). Therefore, we concluded that both the *BjA06.GL1* and *BjB02.GL1* genes are responsible for the leaf trichome trait in mustard.

### Comparative sequence analysis of the candidate genes *BjA06.GL1* and *BjB02.GL1*

**Figure 4:**
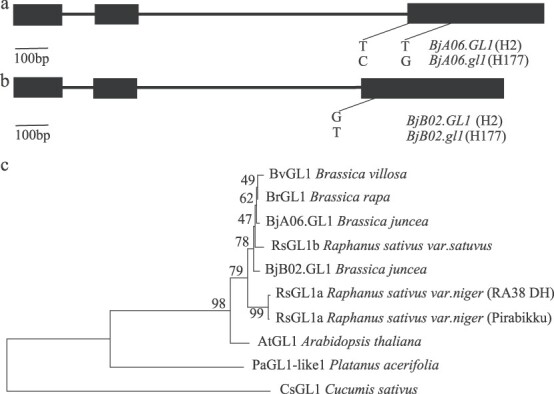
Gene structure and phylogenetic analysis of BjA06.GL1 and BjB02.GL1. a. Gene structure of *BjA06.GL1* and polymorphisms between *BjA06.GL1* from H2 and *BjA06.GL1* from H177. b. Gene structure of *BjB02.GL1* and polymorphisms between *BjB02.GL1* from H2 and *BjB02.GL1* from H177. c. Phylogenetic analysis of BjA06.GL1 and BjB02.GL1 and their homologs in *Brassica*, *P. acerifolia*, and *Cucumis sativus*.

Then, we amplified and comparatively analyzed the genomic and coding sequences of the *BjA06.GL1* and *BjB02.GL1* genes from the H2 and H177 plants, respectively. The coverage of the *BjA06.GL1* and *BjB02.GL1* genes was 59%, and the similarity was 93.07% (Fig. S4a). Sequence analysis revealed that both the *BjA06.GL1* and *BjB02.GL1* genes were composed of three exons. Sequence analysis revealed that two SNPs were present between the two alleles of the *BjA06.GL1* gene from the leaf trichomes parent H2 and the glabrous parent H177, which resulted in two amino acid changes, at positions 92 (tryptophan to tyrosine) and 135 (tyrosine to aspartic acid) (Fig. 4a). Only one SNP existed between the two alleles of the *BjB02.GL1* gene, which resulted in an amino acid change from glycine to valine at the position of 101 (Fig. 4b). The amino acid sequences of BjA06.GL1 and BjB02.GL1 were 226 and 233 residues in length, respectively. BjA06.GL1 and BjB02.GL1 from H2 shared 90.52% amino acid sequence similarity (Fig. S4b). BjA06.GL1 shared 73.42% amino acid sequence similarity with BrGL1. BjB02.GL1 shared 72.15% amino acid sequence similarity with BrGL1 (Fig. S5). The protein sequences of BjA06.GL1 and BjB02.GL1 were used to identify their homologous sequences. BjA06.GL1 and BjB02.GL1 were located in the same clade as other trichome-related MYB genes in *Brassica* (Fig. 4c). These results revealed that both BjA06.GL1 and BjB02.GL1 encodes a putative R2R3-MYB transcription factor in *B. juncea*.

### Transcript expression analysis of *BjA06.GL1* and *BjB02.GL1* genes

**Figure 5:**
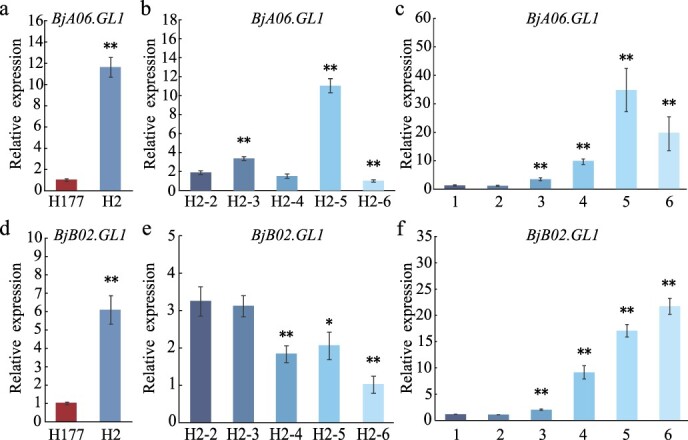
Relative transcript expression levels of *BjA06.GL1* and *BjB02.GL1.* a. Expression levels of *BjA06.GL1* between the leaves with trichomes from H2 and glabrous leaves from H177. b. Relative expression of the *BjA06.GL1* gene from five true leaves (from the second to sixth true leaves) from H2. c. Expression levels of *BjA06.GL1* among different tissues from H2. d. Expression levels of *BjB02.GL1* between the leaves with trichomes from H2 and the glabrous leaves from H177. e. Relative expression of the *BjB02.GL1* gene from five true leaves (from the second to sixth true leaves) from H2. f. Expression levels of *BjB02.GL1* among different tissues from H2. 1–3 represent the roots, stems, and young leaves, respectively, from H2 at the seedling stage. 4–6 represent the stems, old leaves, and flower buds from H2 at the flowering stage. Asterisks indicate significant differences (Student’s *t-*test, ** *P* < 0.01).

Quantitative real-time polymerase chain reaction (qRT-PCR) was used to further assay the transcript expression levels of the *BjA06.GL1* and *BjB02.GL1* genes between leaves with trichomes and glabrous leaves. The transcript expression levels of *BjA06.GL1* and *BjB02.GL1* genes in H2 were significantly greater than those in H177 (Fig. 5a, d). Phenotypic analysis revealed that with increasing leaf size, the density of leaf trichomes on the leaves of the parent H2 decreased. As the density of leaf trichomes in H2 decreased, the relative transcript expression of *BjA06.GL1* gene in the fifth true leaf was significantly greater than that in the other leaves (Fig. 5b). The relative transcript expression of the *BjB02.GL1* gene decreased gradually (Fig. 5e). We further analyzed the transcript expression of the *BjA06.GL1* and *BjB02.GL1* genes among different tissues by qRT-PCR. The *BjA06.GL1* and *BjB02.GL1* genes displayed higher expression levels in the old leaves and flower buds when compared with them from young leaves, stems, and roots (Fig. 5c, f). These results indicate that both the *BjA06.GL1* and *BjB02.GL1* genes coregulate leaf trichome formation in *B. juncea*.

### Knock out of *BjA06.GL1 and BjB02.GL1* reduced leaf trichomes in *B. juncea*

**Figure 6:**
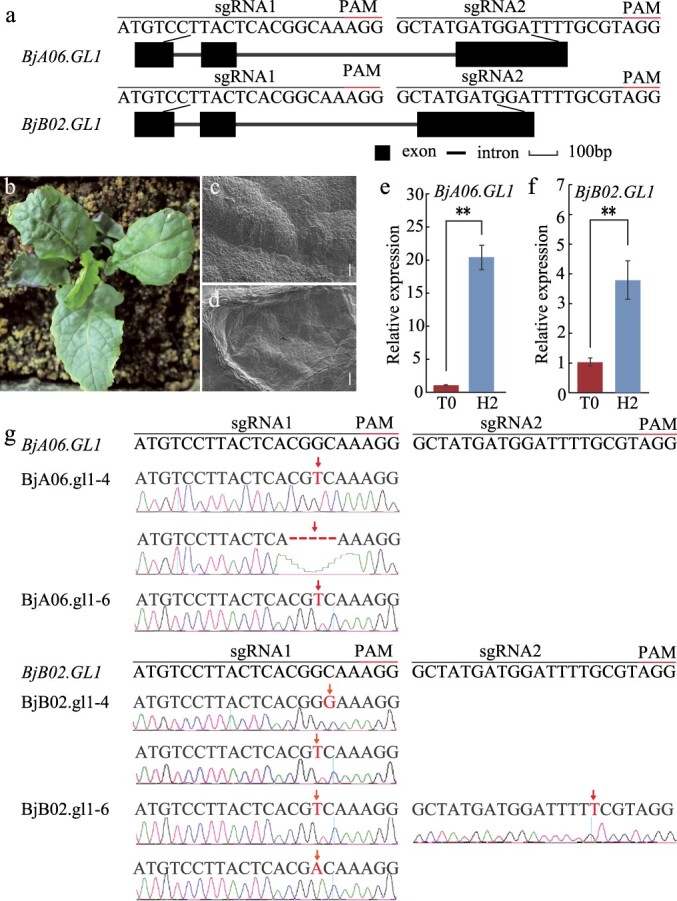
CRISPR/Cas9-mediated gene editing of the *BjA06.GL1* and *BjB02.GL1* genes. a. Target sgRNA selection for *BjA06.GL1* and *BjB02.GL1*. CRISPR/Cas9-targeted sites were selected within exon 1 (sgRNA1) and exon 3 (sgRNA2) of the *BjA06.GL1* and *BjB02.GL1* genes. b. H2 plants with the *BjA06.GL1* and *BjB02.GL1* genes edited had glabrous leaves. c. SEM analysis of the adaxial epidermal leaves from T0 gene-edited plants. d. SEM analysis of the abaxial epidermal leaves from T0 gene-edited plants. e. Expression levels of *BjA06.GL1* between the leaves from T0 plants with glabrous leaves and those from H2 plants with trichomes. f. Expression levels of *BjB02.GL1* in leaves from T0 plants with glabrous leaves and H2 with trichomes. Asterisks indicate significant differences (Student’s *t-*test, ** *P* < 0.01). g. Site-specific mutations of the *BjA06.GL1* and *BjB02.GL1* genes induced by two gRNAs. The PAM is denoted by an overbar. Deletions and insertions are shown in red.

To further verify the gene function of the *BjA06.GL1* and *BjB02.GL1* genes in *B. juncea*, we generated a CRISPR/Cas9 construct that simultaneously targeted the first and third exons of both the *BjA06.GL1* and *BjB02.GL1* genes (Fig. 6a). The hypocotyls from the H2 parent line were used as explants (Fig. S6a). After Agrobacterium-mediated genetic transformation with callus induction and differentiation (Fig. S6, c, d, e), a total of seven T0 transgene-positive plantlets were obtained. Fluorescence screening (Fig. S6c) and PCR analysis (Fig. S6f) further confirmed that the seven transgenic plantlets were positive. The transgene-positive plantlets presented glabrous leaves, and no leaf trichomes could be found on the leaf surface of the gene-edited plants (Fig. 6b, c, d). The transcript expression level of the *BjA06.GL1* and *BjB02.GL1* genes in the leaves of the T0 gene-edited plants were significantly lower than those in the leaves of the H2 plants (Fig. 6e, f). Transcript expression analysis revealed that the *BjA06.GL1* and *BjB02.GL1* genes were more highly expressed in leaves with trichomes than in glabrous leaves. Sanger sequencing analysis revealed that both the *BjA06.GL1* and *BjB02.GL1* genes targeted by sgRNAs caused mutations in both sgRNA targets, which resulted in SNP insertion and presumably caused nonfunctional proteins (Fig. 6 g). CRISPR/Cas9-mediated gene editing of both *BjA06.GL1 and BjB02.GL1* changed the leaf trichome phenotype to a glabrous phenotype.

### Mustard leaf trichomes exhibited greater resistance to aphids

**Figure 7:**
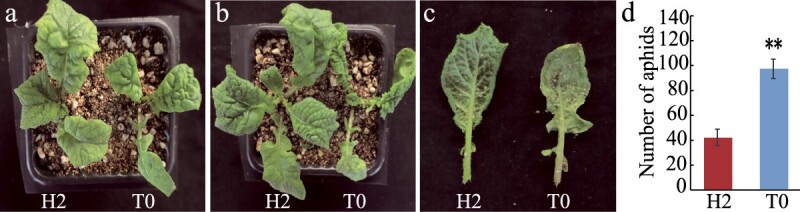
Aphid resistance of leaves with trichomes. a. Growth of CRISPR/Cas9 gene-edited T0 plants and H2 mustard plants before aphid infestation. b. Growth of CRISPR/Cas9 gene-edited T0 plants and H2 mustard plants 7 days after aphid infestation. c. Aphid distribution on the second true leaf of the CRISPR/Cas9 gene-edited T0 and H2 mustard plants 7 days after aphid infestation. d. The number of aphids between the CRISPR/Cas9 infested T0 plants and the H2 parent line. Asterisks indicate significant differences (Student’s *t-*test, ** *P* < 0.01).

Aphid numbers were recorded from 10 different mustard lines in the experimental field. Among them, two leaf mustard plants with leaf trichomes had fewer aphids than the other eight leaf mustard plants with glabrous leaves (Fig. S7a). In the greenhouse, aphid numbers from the parent line H2 with trichomes were greater than those from the parent line H177 with glabrous leaves (Fig. S7b). The H2 parent line and the gene-edited T0 plants were subsequently planted together (Fig. 7a). Seven days after aphid inoculation, the leaf size of the H2 parent line was greater than that of the gene-edited T0 plants (Fig. 7b). The number of aphids on the leaves of H2 with leaf trichomes was also lower than that on the leaves of the gene-edited T0 plants with entirely glabrous leaves (Fig. 7c, d). The aphid numbers on leaves with trichomes were significantly lower than those on leaves with a glabrous surface. In conclusion, leaf trichomes can increase the resistance of mustard to aphids.

## Discussion

Trichomes are a type of trichoid formed by the differentiation of epidermal cells and are found in many different plants. Plant trichomes have multiple protective effects, such as reducing transpiration, protecting against ultraviolet radiation, and conferring insect resistance. To date, trichome formation and variation have been extensively studied in different plants [12]. Plant trichomes are regulated by a complex gene regulatory network. The MYB-bHLH-TTG activators complex and other inhibitors could fine tune their target genes to regulate trichome initiation and development. In *Arabidopsis*, the hormonal signals gibberellin (GA) and jasmonate (JA) synergistically regulate trichome formation. DELLAs could act upstream of the core MYB-bHLH-TTG complex to repress GA-mediated trichome formation [16]. JA-ZIM-domain (JAZ) proteins can repress the transcriptional of bHLH-MYB members of the MYB-bHLH-TTG complex to inhibit trichome formation [25]. In addition to transcription factors and hormones, trichome formation is also regulated by epigenetic regulators [26]. The miR156/SPL/miR172/AP2-like pathway represses the GL1 transcription to delay *Arabidopsis* abaxial leaf trichome initiation in the juvenile phase [19]. Research on functional genes related to plant trichomes has provided valuable insights into the molecular mechanisms underlying trichome development.

The MYB transcription factor was predicted to be a candidate gene to regulate leaf trichome formation. The *AtGL1* gene encodes an MYB transcription factor that interacts with AtGL3 and AtTTG1 to regulate trichome differentiation and morphogenesis [15]. In *B. rapa*, the trichome trait is controlled by the dominant gene *BrGL1*, which is located on chromosome A06 [27]. The new allele of *BrTRI1* (BraA06g038610.3.5C) resulted in a trichomeless phenotype [23]. In *B. napus*, constitutive expression of *BnaA.GL1.a* (BnaA06g31780D) in *Arabidopsis* induced trichome formation on both leaves and stems [22]. Two *PaGL1*-like genes regulate trichome development in the London plane (*Platanus acerifolia*) [28]. The *GL1* orthologues in *B. rapa* and *Raphanus sativus* might contribute to leaf trichome variation [29]. CRISPR/Cas9-mediated knockout of *RsGL1a* and *RsGL1b* caused the hairless phenotypes in radish [24]. We found that both the *BjA06.GL1* and *BjB02.GL1* genes regulate leaf trichome development in leaf mustard. However, the expression pattern of *BjA06.GL1* was different from the expression pattern of *BjB06.GL1.* This may be because the promoter sequences of the two homologous genes *BjA06.GL1* and *BjB02.GL1* are completely different. Moreover, the two *BjGL1* homologous genes displayed differential effects on trichome patterning. In addition, knocking out both the *BjA06.GL1* and *BjB02.GL1* genes changed leaves with trichomes to glabrous leaves. Thus, these orthologous *GL1* genes contribute to leaf trichome variation in *Brassicaceae* plants.

In *B. juncea*, the SV located in the BjuVB02G54610 gene was found to be responsible for leaf trichome development [5]. Since the draft genome sequence of the vegetable *B. juncea* T84–66 was sequenced [2], oil-type and leaf-type mustards, including Varuna [30], AU213 [31], SY [32], and XC [33], have also been sequenced. The sequenced genome of *B. juncea* has helped us rapidly identify the haplotype differences in candidate genes. In this study, a natural LTR inserted into *BjB02.GL1* was identified after a comparative analysis of the gene among different sequenced *B. juncea* genomes. Blast analysis revealed that the SV sequence insertion in the second exon of *BjB02.GL1* gene was a copia LTR insertion. This result further confirmed that the SV is not completely correlated with leaf trichome variation in natural populations [5]. In our study, genetic analysis indicated that the leaf trichome formation in *B. juncea* is a quantitative trait. The *BjB02.GL1* and *BjA06.GL1* genes determine in large part of the leaf trichome variation in *B. juncea.* Molecular markers of the *BjA06.GL1* and *BjB02.GL1* explained >97.18% of the leaf trichome variation in the F2 segregating population. The two molecular markers together with the LTR in *BjB02.GL1* could explain the variation in leaf trichomes. However, several plants with leaf trichomes in the F2 segregating population were not associated with the *BjA06.GL1* and *BjB02.GL1* genes. Apart from the *BjB02.GL1* and *BjA06.GL1* genes, other genes could also be involved in trichome variation in *B. juncea*. This result indicates that the leaf trichomes are not regulated by a single gene and that the genetic inheritance of the trichome trait in *B. juncea* is complex. Further exploration of other genes and gene regulation networks could expand our understanding of leaf trichome variation in *B. juncea.*

Leaf trichomes can alleviate plant responses to biotic and abiotic stresses. Trichomes not only act as a physical barrier to prevent herbivory but also affect the taste and quality of vegetables. In *B. napus*, leaf trichomes can determine aphid feeding preference [34]. Transcriptomic analysis revealed that the glandular trichomes and their secreted chemical compounds confer strong resistance to aphids in tomatoes [35]. In wheat, the biosynthesis of the defense metabolites provides a stronger defense than leaf trichomes against *Rhopalosiphum padi* aphids [36]. In our research, leaf trichomes presented greater resistance to aphids in leaf mustard. In addition to physical inhibition by trichomes, whether chemical compounds provide a defense against aphids needs to be further studied. Our study could improve the understanding of the molecular mechanism of the leaf trichome formation and accelerate the breeding of insect-resistant mustard. The molecular mechanism underlying leaf trichome formation in *B. juncea* needs to be fully elucidated.

## Conclusions

In this study, both the *BjA06.GL1* and *BjB02.GL1* genes, which encode R2R3-MYB transcription factors, were cloned. Both of these genes were significantly more highly expressed in mustard plants with leaf trichomes than in those with glabrous leaves. Gene editing of both *BjA06.GL1* and *BjB02.GL1* changed leaves with trichomes to glabrous leaves. Leaf trichomes increase the leaf resistance to aphids in *B. juncea*.

## Materials and methods

### Plant materials, phenotype observation, and quantitative measurement of trichomes

The different mustard inbred lines with or without leaf trichomes used for gene haplotype detection and gene mapping are listed in Table S4. Among them, the leaves from the XC, H10, H177, Varuna, and SY inbred lines were glabrous. The leaves from the 102B, H58, H2, and T84–66 inbred lines presented trichomes. The inbred line with leaf trichomes (H2) and the inbred line with glabrous leaves (H177) were used as the two parental lines. Then, H2 was crossed with H177 to produce the F1 population, and the F1 plants were self-pollinated to produce the F2 population in 2022. The parental lines together with the F1 and F2 populations were used to investigate the genetic inheritance pattern and mapped genes related to leaf trichomes. All of the mustard plants used in this study were planted in the experimental plots at Xinyang Normal University (Xinyang, China). The trichome number was calculated by photographing the second leaf. ImageJ software was subsequently used to count the number of trichomes in a given area. The trichome density was calculated in triplicate. SEM was used to examine the adaxial and abaxial properties of the leaves from H2 and H177 as previously described [37].

### Genome resequencing and identification of candidate regions

Leaves with trichomes bulks (LTBs) and leaves with glabrous bulks (LGBs) were collected by pooling 30 young leaves with trichomes and 30 glabrous leaves selected from the F2 segregation population. Samples from the two parental lines and two F2 segregating pools were used to extract genomic DNA. Resequencing of the four DNA pools was performed with the Illumina NovaSeq 6000 platform by Biomarker Technologies (Beijing, China).

The raw sequencing data were filtered to remove low-quality data. The quality-controlled sequencing data were subsequently aligned to the XC reference genome via Burrows–Wheeler Aligner (BWA) software, and the alignment results were converted to BAM files via SAMtools. SNPs and indel in each sample were detected with GATK software, and further filtered with the parameters ‘QD < 2.0 || MQ < 40.0 || FS > 60.0 || SOR > 3.0 || MQRankSum < −12.5 || ReadPosRankSum < −8.0’. Δ(SNP/indel index) analysis was performed with the SNP index for different loci. QTL-seq was used to identify candidate intervals with a window size of 1 Mb and a step size of 100 kb [38]. The resulting variant genes were then functionally annotated via ANNOVA software. The 95% and 99% confidence levels were selected as the screening thresholds. G’ values [39] and the two-tailed Fisher exact test [40] were also used to identify potential QTL intervals. The overlapping interval of these three methods was considered as the final QTL region.

### Screening and comparative analysis of gene sequence of *BjA06.GL1* and *BjB02.GL1*

Three strategies were used to screen candidate genes for leaf trichomes in *B. juncea*. First, we focused on SNPs in the QTL regions between the two parents. Second, candidate genes related to leaf trichomes were selected on the basis of their functional annotations on the leaf mustard XC genome. Third, molecular markers were developed to further confirm the relationship between the haplotype of the candidate genes and the leaf trichome phenotype. Finally, the candidate genes controlling leaf trichomes were obtained. The gene-specific primers used for *BjA06.GL1* and *BjB02.GL1* were used to amplify the gene sequences from the two parental lines separately. The PCR products were subsequently subcloned and inserted into the pMDTM19-T vector (Takara). They were sequenced via Sanger sequencing. SeqMan was used for sequence alignment. Gene-specific molecular markers, which were developed on the basis of SNP and indel information, were used to distinguish different haplotypes.

### Phylogenetic analysis of BjA06.GL1 and BjB02.GL1

The amino acid sequence of the AtGL1 protein from *Arabidopsis thaliana* was used as a query to identify GL1 homologous proteins from the NCBI database (https://www.ncbi.nlm.nih.gov/). MEGA 7 (https://www.megasoftware.net/) was used for the phylogenetic analysis of BjA06.GL1 and BjB02.GL1 in *B. juncea* via the neighbor-joining method with 1000 bootstrap replications. The conserved domains of BjA06.GL1 and BjB02.GL1 were determined with MEGA 7 and GeneDoc. The GenBank accession numbers of the GL1 homologous proteins are provided in Table S5.

### qRT-PCR analysis of *BjA06.GL1* and *BjB02.GL1*

The seeds of the two inbred lines H2 and H177 were sown at 25°C (16 h of light and 8 h of darkness per day) in a light incubator (GXZ-type illumination cultivation incubator). After 33 days of growth, leaves from H2, H177, and T0–4 were collected separately. The SAM and the first, second, third, fourth, fifth, and sixth leaves from plants with leaf trichomes (H2) were sampled. The roots, stems, and young leaves were collected at the seeding stage of H2. The stems, old leaves, and flower buds at the flowering stage of H2 were also collected. The samples were frozen with liquid nitrogen and stored at −80°C until use.

Total RNA was extracted with a MiniBEST Plant RNA Extraction Kit (TaKaRa No9769). cDNA was synthesized by using a PrimeScriptTM 1st Strand cDNA Synthesis Kit (Code No: 6110A). qRT-PCR of *BjA06.GL1* and *BjB02.GL1* was carried out on the ABI PRISM 7300 Real-Time PCR System (Applied Biosystems). The *BjActin* gene was used as an internal control [1]. The transcript expression levels of *BjA06.GL1* and *BjB02.GL1* were further analyzed via the 2^-ΔΔCT^ method [41]. The primers used for qRT-PCR are listed in Table S6.

### CRISPR/Cas9 vector construction and *Agrobacterium tumefaciens*-mediated leaf mustard transformation

Two target sgRNA sequences for *BjA06.GL1 and BjB02.GL1* were designed with CRISPR-P 2.0 [42]. Two AtU6 promoter-sgRNA-AtU6 terminator cassettes were amplified with a pCBC-DT1T2 vector as a template [43]. The PCR fragments were inserted into the pKSE401G vector [44] and confirmed by Sanger sequencing. The mustard inbred line H2, with leaf trichomes, served as a wild-type strain for CRISPR/Cas9-mediated mutagenesis. The previously established Agrobacterium-mediated gene editing genetic transformation system for *B. juncea* was used [33]. First, seeds of the inbred line H2 were soaked in 75% ethanol for 1 min. Then, they were sterilized for 5 min in a 0.15% HgCl₂ solution and washed three times with sterile ddH_2_O. The hypocotyls were obtained from seeds planted for ~5–7 days in the dark with MS medium. After coculture, callus induction, selective culture, and root generation, the mustard mutants were first screened by GFP fluorescence, which was examined by using a fluorescent protein excitation light source with a GFP filter (LUYOR-3415RG). SEM was further used to examine the adaxial and abaxial leaves of the T0 plants. The CRISPR/Cas9-mediated gene-edited plants were identified via PCR amplification and DNA sequencing.

### Aphid resistance assay

First, the number of aphids (*Lipaphis erysimi*) on different mustard inbred lines was determined with three replications in the experimental field of Xinyang Normal University in 2023. Second, the two parent lines, H2 and H177, were planted in a greenhouse with 16 h/8 h (light/dark, photoperiod) at 25°C. Three weeks later, aphids were inoculated on the leaves of both parent lines, with three biological replicates each. Finally, the aphids were further allowed to infest the leaves of the CRISPR/Cas9 gene-edited T0 plants and plants of the H2 parent line with leaf trichomes. The number of aphids was counted 7 days after inoculation. The aphid resistance assay in the greenhouse involved confinement to a cage with an insect-proof net with three replications.

## Supplementary Material

Web_Material_uhae314

## Data Availability

The data used in this study are available in the Genome Sequence Archive of the National Genomics Data Center and can be accessed with CRA017217.
