# Supplementary material for: Two novel alleles of the MYB transcription factor BjA06.GL1 and BjB02.GL1 control leaf trichomes and enhance resistance to aphids in Brassica juncea
Source: Hortic Res. 2024 Nov 12;12(2):uhae314. doi: 10.1093/hr/uhae314 (PMC11879403; doi:10.1093/hr/uhae314)
Supplement: Web_Material_uhae314 [file web_material_uhae314.zip › Fig. S5.pdf]

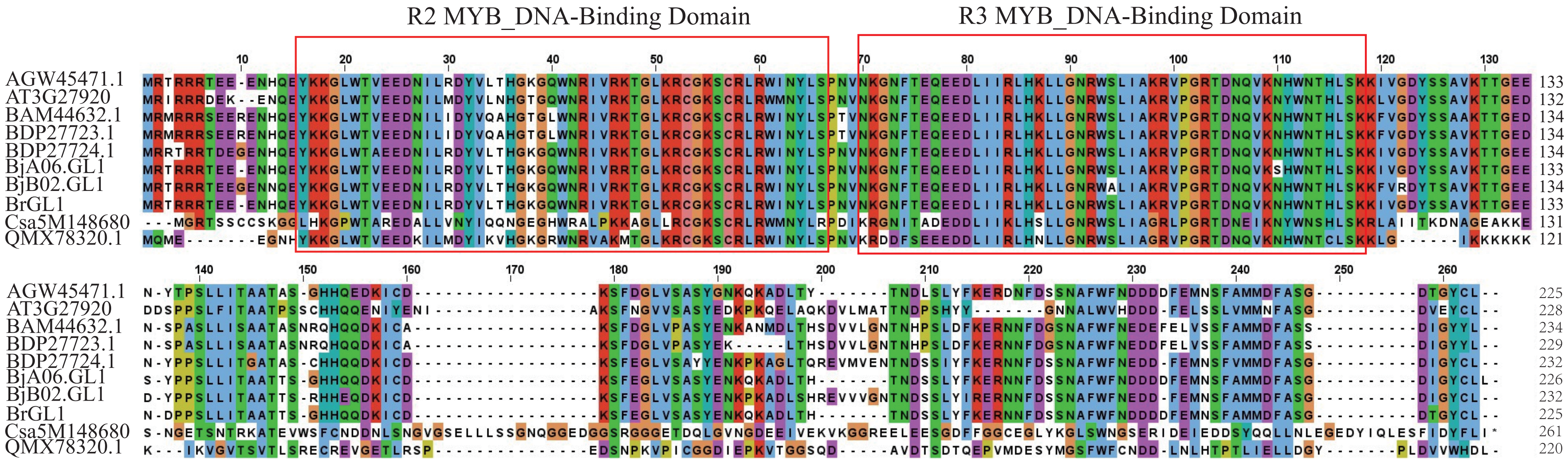
R2 MYB_DNA-Binding Domain
R3 MYB_DNA-Binding Domain
AGW45471.1
AT3G27920
BAM44632.1
BDP27723.1
BDP27724.1
BjA06.GL1
BjB02.GL1
BrGL1
Csa5M148680
QMX78320.1
