# Supplementary figures and images for: Two novel alleles of the MYB transcription factor BjA06.GL1 and BjB02.GL1 control leaf trichomes and enhance resistance to aphids in Brassica juncea
Source: Hortic Res. 2024 Nov 12;12(2):uhae314. doi: 10.1093/hr/uhae314 (PMC11879403; doi:10.1093/hr/uhae314)

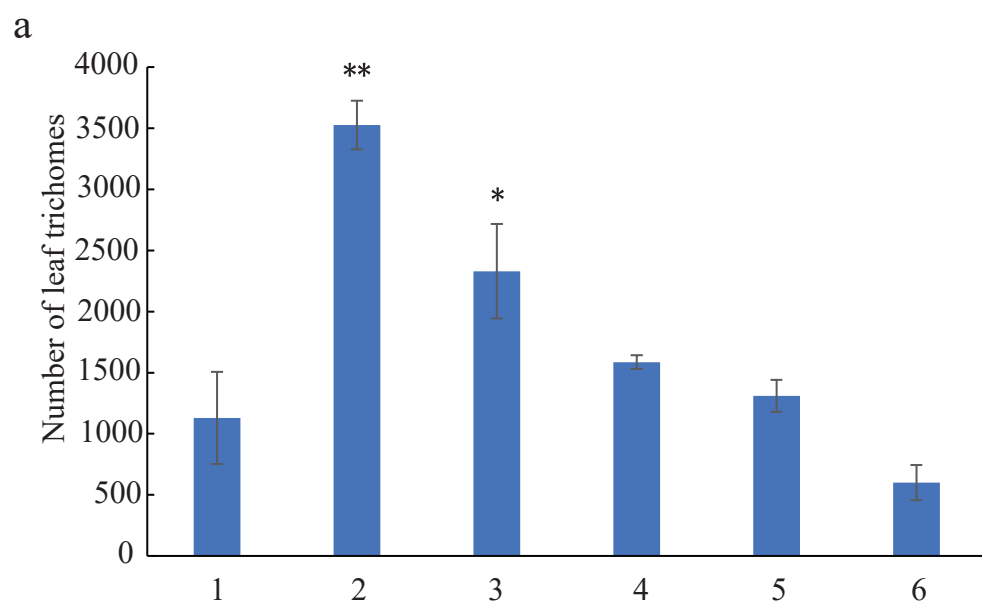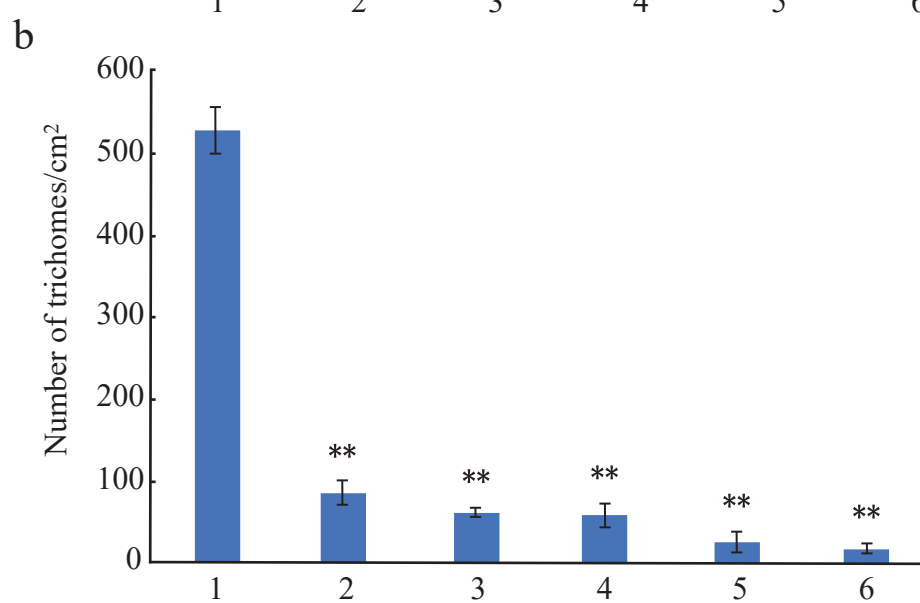

Supplement: Web_Material_uhae314 [file web_material_uhae314.zip › Fig. S1.pdf]

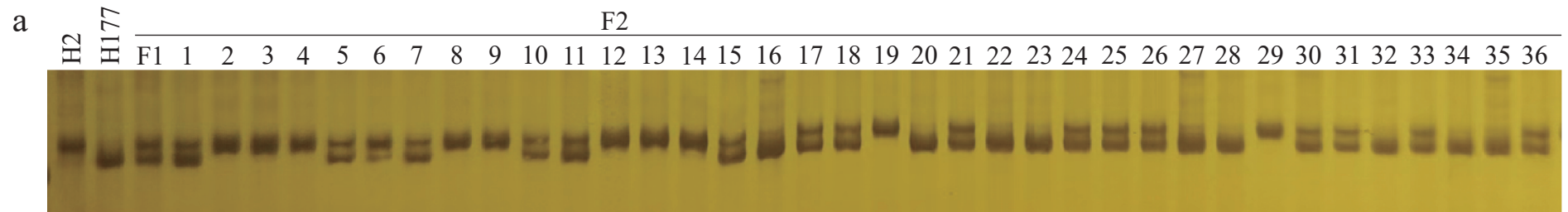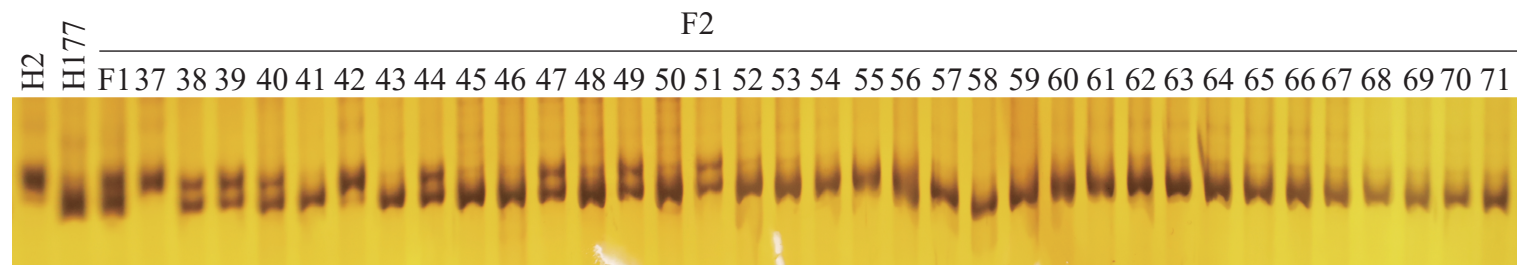

*BjA06.GL1*

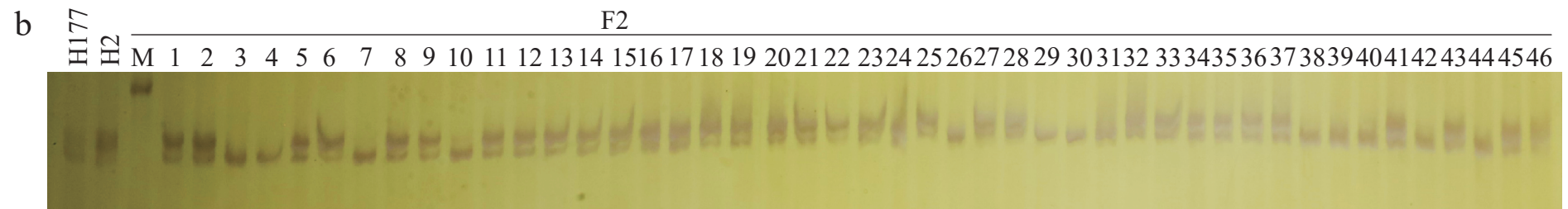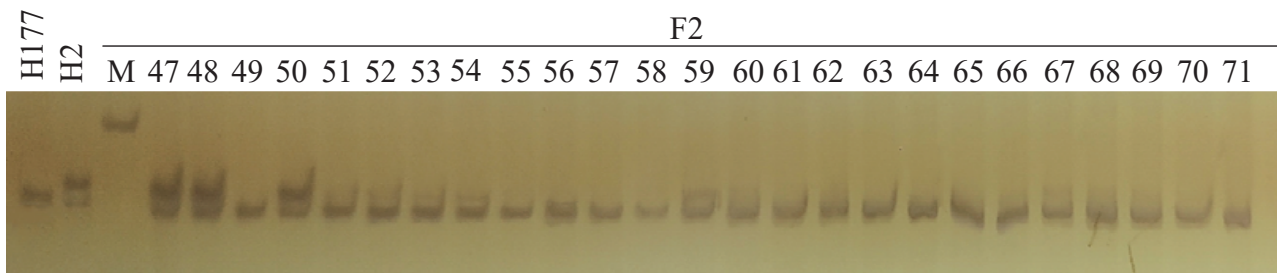

*BjB02.GL1*

Supplement: Web_Material_uhae314 [file web_material_uhae314.zip › Fig. S2.pdf]

a

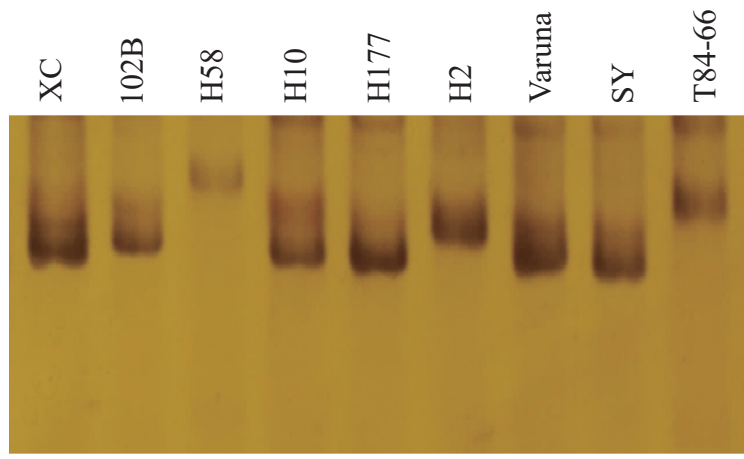

b

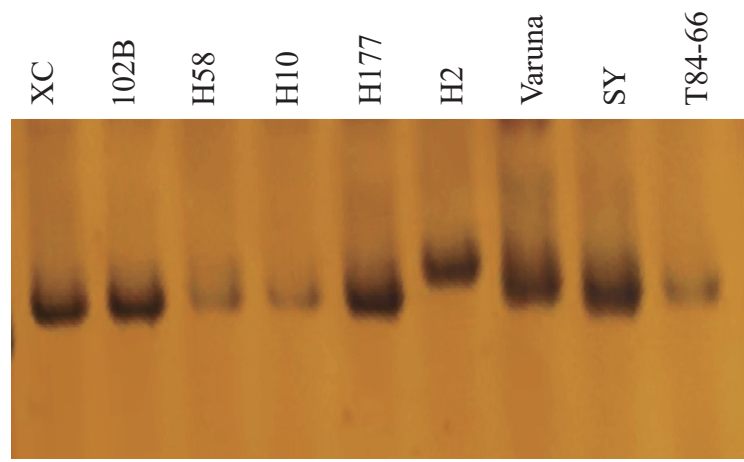

Supplement: Web_Material_uhae314 [file web_material_uhae314.zip › Fig. S3.pdf]

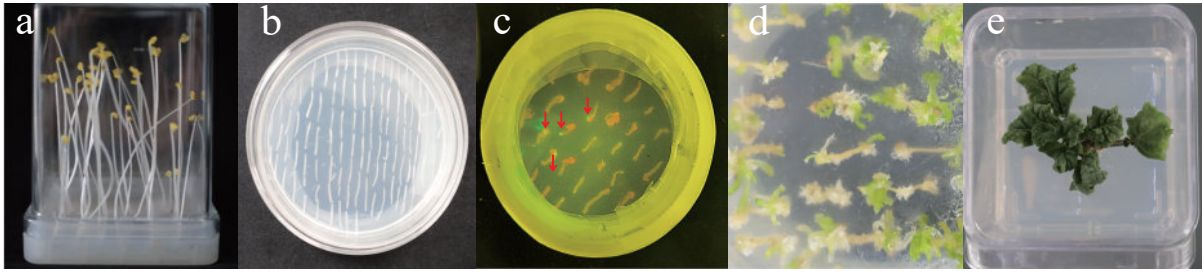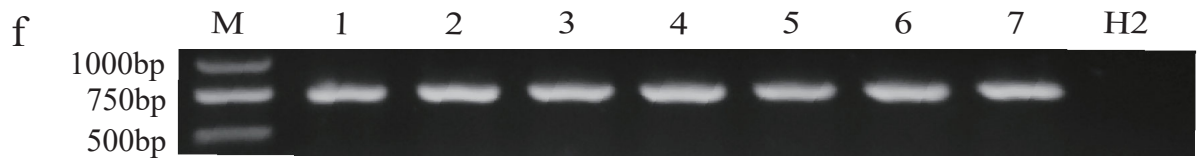

Supplement: Web_Material_uhae314 [file web_material_uhae314.zip › Fig. S6.pdf]

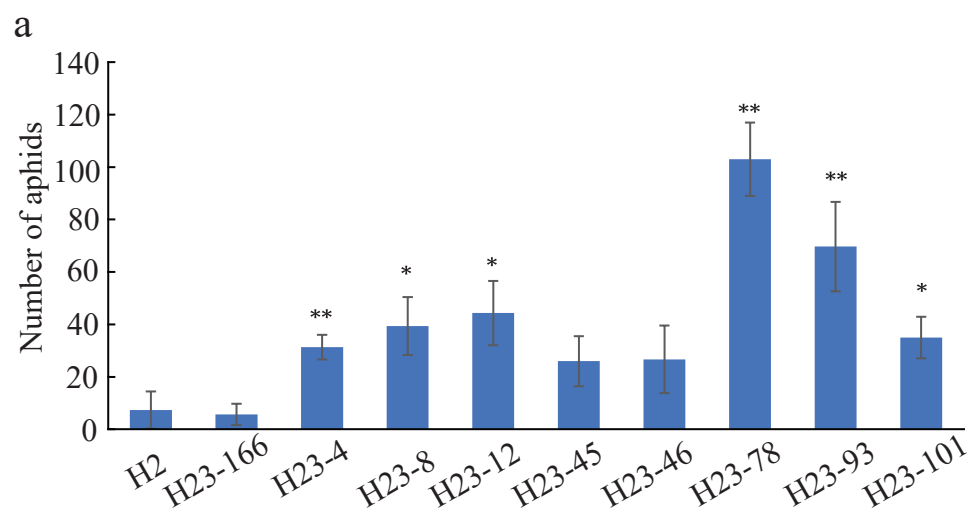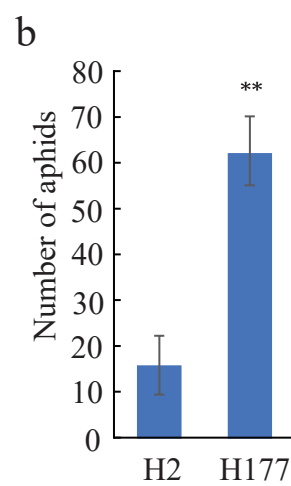

Supplement: Web_Material_uhae314 [file web_material_uhae314.zip › Fig. S7.pdf]
